# Patterns and implications of 2025 NIH-F31 grant terminations for the predoctoral training pipeline

**DOI:** 10.1093/haschl/qxag065

**Published:** 2026-03-18

**Authors:** Jahn Jaramillo, Audrey Harkness

**Affiliations:** Department of Public Health Sciences, University of Miami, Miami, FL 33136, United States; School of Nursing and Health Studies, University of Miami, Miami, FL, United States

**Keywords:** students, graduate, minority groups, predoctoral training, research support as topic, grant termination, research workforce diversity, National Institutes of Health, Health Equity

## Abstract

**Introduction:**

In 2025, the National Institutes of Health (NIH) terminated student grants across the Ruth L. Kirschstein National Research Service Award Individual Predoctoral Fellowship mechanisms, including both general F31 and F31-Diversity awards, disrupting a critical training pipeline marked by inequities. Since the terminations, the status of many grants has shifted amid an evolving landscape of freezes, appeals, and reinstatements, leading to prolonged uncertainty for predoctoral trainees.

**Methods:**

In this article, we analyzed from publicly available data the scope and geographical distribution of terminated F31 (general and diversity) awards from 2025 and considered the implications of these terminations on trainees. We queried publicly available data from Grant Witness (November 16, 2025, to December 18, 2025), a website that monitors grant terminations across various US government agencies, such as NIH.

**Results:**

We found that 405 F31 grants were affected by the 2025 terminations; of these, 136 were general F31s, and 269 were F31-Diversity awards. States in the South and Midwest were disproportionately represented among the terminations of diversity-promoting F31s.

**Conclusion:**

Federal agencies and academic institutions may consider implementing safeguards, including protections against midyear grant terminations and emergency bridge funding to protect trainees during periods of political and funding instability.

Key Points

- NIH predoctoral fellowships strengthen US research capacity by improving scientific productivity, supporting innovative research, and developing the next generation of investigators.
- In this brief report, we analyze 2025 NIH F31 grant terminations and find disproportionate impacts on diversity-focused fellowships and geographic clustering in the South and Midwest.
- Policy measures are needed to ensure training continuity and career development for predoctoral trainees.

## Introduction

The National Institutes of Health (NIH) has, for over 50 years, awarded predoctoral fellowships, typically under their F31 mechanism known as the Ruth L. Kirschstein National Research Service Award (NRSA) Individual Predoctoral Fellowship [Parent F31].^1,2^ The F31 supports doctoral trainees’ scholarship in health research, with an accompanying set of training and mentoring goals to prepare them to become independent NIH-funded investigators addressing priority national health issues.^1^ Recognizing the historical and persistent inequities across the biomedical research training pipeline that prevent qualified individuals from pursuing scientific careers, which include limited access to training opportunities, mentors and professional networks, and socioeconomic barriers,^2-4^ the NIH established the Ruth L. Kirschstein NRSA Individual Predoctoral Fellowship to Promote Diversity in Health-Related Research [Parent F31-Diversity]. The only difference between the general F31 and the diversity-promoting F31 is that the latter was designed to address systemic barriers to securing early-career funding for underrepresented scholars in the biomedical, clinical, behavioral, and social sciences;^4,5^ as with the general F31, the F31-Diversity was only awarded to support exceptional science and scholars.^6^ Under the now-expired NIH F31-Diversity funding opportunity announcement,^7^ the NIH defined underrepresented scholars as individuals from racial and ethnic groups (ie, African Americans, Hispanics or Latinos, American Indians or Alaska Natives, Native Hawaiians, and other Pacific Islanders), persons with disabilities, and those from disadvantaged backgrounds. Individuals applying to the F31, regardless of mechanism, undergo a rigorous and competitive NIH application process, including developing a robust application, scientific review from three reviewers, and then a full panel study section, and finally, NIH council review before funding decisions are made.^7^ F31 awardees under both opportunities have made substantial contributions to biomedical and behavioral health science, including but not limited to the science of health disparities and equity research to date.^2,8^

In 2025, hundreds of awarded F31 grants were terminated pursuant to Executive Order 14151, which resulted in the discontinuation of federal funding for Diversity, Equity, and Inclusion (DEI) initiatives.^9^ According to recent court filings,^10^ the NIH indicated that DEI and gender identity grants were “low-value and off-mission,” were “used to support unlawful discrimination on the basis of race and other protected characteristics,” and relied “on artificial and nonscientific categories,” and were thus deemed incompatible with agency priorities (under 2 CFR § 200.340).^11^ Of the 2282 NIH grants recently terminated (according to available published figures from June 2025 by the Association of American Medical Colleges), 38% were research training and career development awards, representing more than $512 million in lost funding. Nearly one-third of terminated grants were F31 predoctoral fellowships.^12^ Given that only 10% of NIH predoctoral awards (which include F31s) go to trainees from underrepresented groups, and just 1% to Black applicants according to a 2020 *Racism in Science Report* from the NIH Advisory Committee to the Director Working Group,^3^ the allocation of grants does not equivalently reflect US demographics; U.S. Census Bureau estimated Black/African Americans comprised 12% and Latinxs/Hispanics represented 19% of the population in 2020.^13^ This already constricted training pipeline is now narrowing even further for predoctoral students experiencing ongoing uncertainty about terminated grants, funding freezes, and partial reinstatements.^14^ Some terminated grants, including F31s,^15,16^ were restored in June 2025^17^ following the court's decision in *American Public Health Association (APHA) v. NIH*^18^ and *Commonwealth of Massachusetts v. Kennedy, Jr.,*^19^ which determined the terminations as “arbitrary and capricious” and “of no force.”^20^ At the time of writing, the cases are ongoing, and the status of many remaining grants is still pending.^21,22^ As such, in this paper, we present updated findings from a publicly available dataset on F31 terminations as of November 2025 (using F31-G to denote general awards and F31-D to denote diversity awards throughout), and close with recommendations for strengthening protections for predoctoral, early-career researchers.

## Methods

In 2025, hundreds of NIH grants, including F31s, were terminated. Termination letters had language that suggested the projects were unscientific and low-value.^23,24^ Although some literature has described the impact of these NIH grants overall,^12,25^ there has been relatively little focus in the literature on the specific impacts of F31 dissertation grants, which affect students who are in a unique position compared to faculty affected by the broader terminations. To address this gap in the literature, we provide a snapshot of the current grant-termination landscape based on publicly available data and explore the implications of these terminations for student training.

To provide an overall snapshot of the F31 terminations, we examined publicly available data from *Grant Witness*, an independent tracking project that compiles NIH grant terminations using government databases, court filings, and submissions from affected researchers (weekly reports noted through October 15^th^, 2025; subsequent update frequency unspecified).^26^  *Grant Witness* is run by a group of researchers and volunteers, led by Scott Delaney and Noam Ross.^26,27^ This database assigns one of five statuses to each terminated grant: terminated (funding ended with no reinstatement), possibly reinstated (typically due to court orders, but not yet confirmed or verified that funding resumed), frozen (funding placed on hold at institutions targeted for freezes), possibly unfrozen (previously frozen grants now showing signs of resumed activity), and unfrozen (confirmed resumed activity). We downloaded the full dataset (ie, CSV file accessed November 16, 2025), filtered for F31 grants by state and termination status. We assessed geographic variation (at the state level) to determine the burden of terminations by state and whether the termination status, area of research, or F31 mechanism affected was concentrated in particular regions, with potential implications for local research capacity. We then manually reviewed each abstract (included in the dataset), and categorized grants into DEI/SGM related (if their primary aims sought to examine or address health inequities in sexual and gender minority populations or targeted other marginalized subpopulations), and non-DEI/SGM related (those with primary aims focused on laboratory or mechanistic-based research in fields such as neuroscience, immunology, or molecular biology). This categorization was conducted to evaluate the types of research affected (and whether grants flagged as “DEI-related” in the publicly available database were indeed DEI-focused or represented other scientific areas), as a preliminary review of abstracts flagged as DEI/SGM-related suggested that some were not specifically focused on DEI/SGM topics, despite this being the stated reason why they were terminated. This approach also allowed us to assess patterns across the intersection of research topic (DEI/non-DEI) and mechanism (F31-G/F31-D), recognizing that DEI/SGM-related research may be supported under either mechanism (F31-D eligibility pertains to the trainee rather than the research topic).

## Results

Out of 5464 terminated grants reported on *Grant Witness*, 405 were predoctoral F31 grants (Table 1). Of those 405 F31 grants, 136 (34%) were awarded under the general F31 mechanism and 269 (66%) were under the F31-Diversity mechanism. Among the 136 general F31s that were terminated, 21 (15%) remained terminated, 92 (68%) were possibly reinstated, 21 (15%) were frozen, 1 (<1%) was possibly unfrozen, and 1 (<1%) was unfrozen. In contrast, among the 269 terminated F31-Diversity awards, 162 (60%) remained terminated, 94 (35%) were possibly reinstated, 12 (4%) were frozen, and 1 (<1%) was possibly unfrozen.

**Table 1.** State-level distribution of FY 2025 F31 terminations by state, F31 grant mechanism, and current termination status category.

State	Total	F31-G only	F31-D only	Still terminated			Possibly reinstated			Frozen funding			Possibly Unfrozen funding^a^		
				F31-G	F31-D	T	F31-G	F31-D	T	F31-G	F31-D	T	F31-G	F31-D	T
1. Massachusetts (MA)	**78**	53	25	—	2	2	53	23	76	—	—	—	—	—	—
2. New York (NY)	**61**	33	28	—	15	15	29	11	40	3	1	4	1	1	2
3. California (CA)	**56**	12	44	10	15	25	2	29	31	—	—	—	—	—	—
4. Illinois (IL)	**35**	18	17	—	8	8	—	—	—	18	9	27	—	—	—
5. Pennsylvania (PA)	**16**	0	16	—	14	14	—	2	2	—	—	—	—	—	—
6. Texas (TX)	**15**	0	15	—	15	15	—	—	—	—	—	—	—	—	—
7. Maryland (MD)	**15**	5	10	4	9	13	1	1	2	—	—	—	—	—	—
8. Florida (FL)	**12**	1	11	1	11	12	—	—	—	—	—	—	—	—	—
9. Georgia (GA)	**10**	2	8	1	7	8	1	1	2	—	—	—	—	—	—
10. North Carolina (NC)	**9**	1	8	1	6	7	—	—	—	—	2	2	—	—	—
11. Tennessee (TN)	**9**	2	7	1	7	8	1	—	1	—	—	—	—	—	—
12. Michigan (MI)	**8**	0	8	—	8	8	—	—	—	—	—	—	—	—	—
13. Wisconsin (WI)	**7**	2	5	1	2	3	1	3	4	—	—	—	—	—	—
14. Rhode Island (RI)	**7**	4	3	—	—	—	3	3	6		—		1	—	1
15. New Mexico (MN)	**6**	0	6	0	1	1	—	5	5	—	—	—	—	—	—
16. Connecticut (CT)	**6**	0	6	—	6	6	—	—	—	—	—	—	—	—	—
17. Virginia (VA)	**5**	0	5	—	5	5	—	—	—	—	—	—	—	—	—
18. Alabama (AL)	**5**	0	5	—	4	4	—	1	1	—	—	—	—	—	—
19. Washington (WA)	**5**	1	4	—	—	—	1	4	5	—	—	—	—	—	—
20. Utah (UT)	**4**	0	4	—	4	4	—	—	—	—	—	—	—	—	—
21. Indiana (IN)	**4**	0	4	—	4	4	—	—	—	—	—	—	—	—	—
22. Colorado (CO)	**4**	0	4	—	—	—	—	4	4	—	—	—	—	—	—
23. South Carolina (SC)	**3**	0	3	—	3	3	—	—	—	—	—	—	—	—	—
24. New Jersey (NJ)	**3**	0	3	—	2	2	—	1	1	—	—	—	—	—	—
25. Missouri (MO)	**3**	0	3	—	3	3	—	—	—	—	—	—	—	—	—
26. Minnesota (MN)	**3**	0	3	—	—	—	—	3	3	—	—	—	—	—	—
27. Oregon (OR)	**2**	0	2	—	—	—	—	2	2	—	—	—	—	—	—
28. Ohio (OH)	**2**	0	2	0	2	2	—	—	—	—	—	—	—	—	—
29. New Hampshire (NH)	**2**	0	2	0	2	2	—	—	—	—	—	—	—	—	—
30. Louisiana (LA)	**2**	0	2	0	2	2	—	—	—	—	—	—	—	—	—
31. Washington D.C. (DC)	**2**	1	1	1	1	2	—	—	—	—	—	—	—	—	—
32. Puerto Rico (PR)	**1**	0	1	—	1	1	—	—	—	—	—	—	—	—	—
33. North Dakota (ND)	**1**	0	1	—	1	1	—	—	—	—	—	—	—	—	—
34. Mississippi (MS)	**1**	0	1	—	1	1	—	—	—	—	—	—	—	—	—
35. Kentucky (KY)	**1**	0	1	0	1	1	—	—	—	—	—	—	—	—	—
36. Arizona (AZ)	**1**	0	1	0	0	0	0	1	1	—	—	—	—	—	—
37. Nebraska (NE)	**1**	1	0	1	0	1	—	—	—	—	—	—	—	—	—
**Total**	**405**	**136**	**269**	**21**	**162**	**183**	**92**	**94**	**186**	**21**	**12**	**33**	**2**	**1**	**3**

This table presents the number of FY 2025 F31 awards in each state and their classification across four funding status categories: Still Terminated, Frozen Funding, Possibly Unfrozen Funding, and Possibly Reinstated. Values are shown separately for general F31 and F31-Diversity awards to reflect differential impacts. For each category, both F31 and F31-Diversity counts are provided, along with state-level totals.

^**a**^Includes both “possibly unfrozen” and “unfrozen” awards, which were collapsed into this category due to very small counts (approximately 2 and 1 awards, respectively).

Source: Authors’ analysis of data from Grant Witness.

Terminated F31 grants were spread across 37 states, and although the impact was national, a large share of grants that remained terminated (*n* = 183) were concentrated in California, New York, and Texas. States that had a larger share of possibly reinstated grants were Massachusetts, New York, and California. States with continued frozen F31 funding included Illinois, New York, and North Carolina. Possibly unfrozen awards were also observed in New York, and fully unfrozen funding was identified in Rhode Island. F31-Diversity grant terminations clustered mostly in the Midwest and the South. For example, several states like Texas, Alabama, Mississippi, Kentucky, and Louisiana had 100% of their terminated awards affecting F31-Diversity recipients.

**Figure 1.**
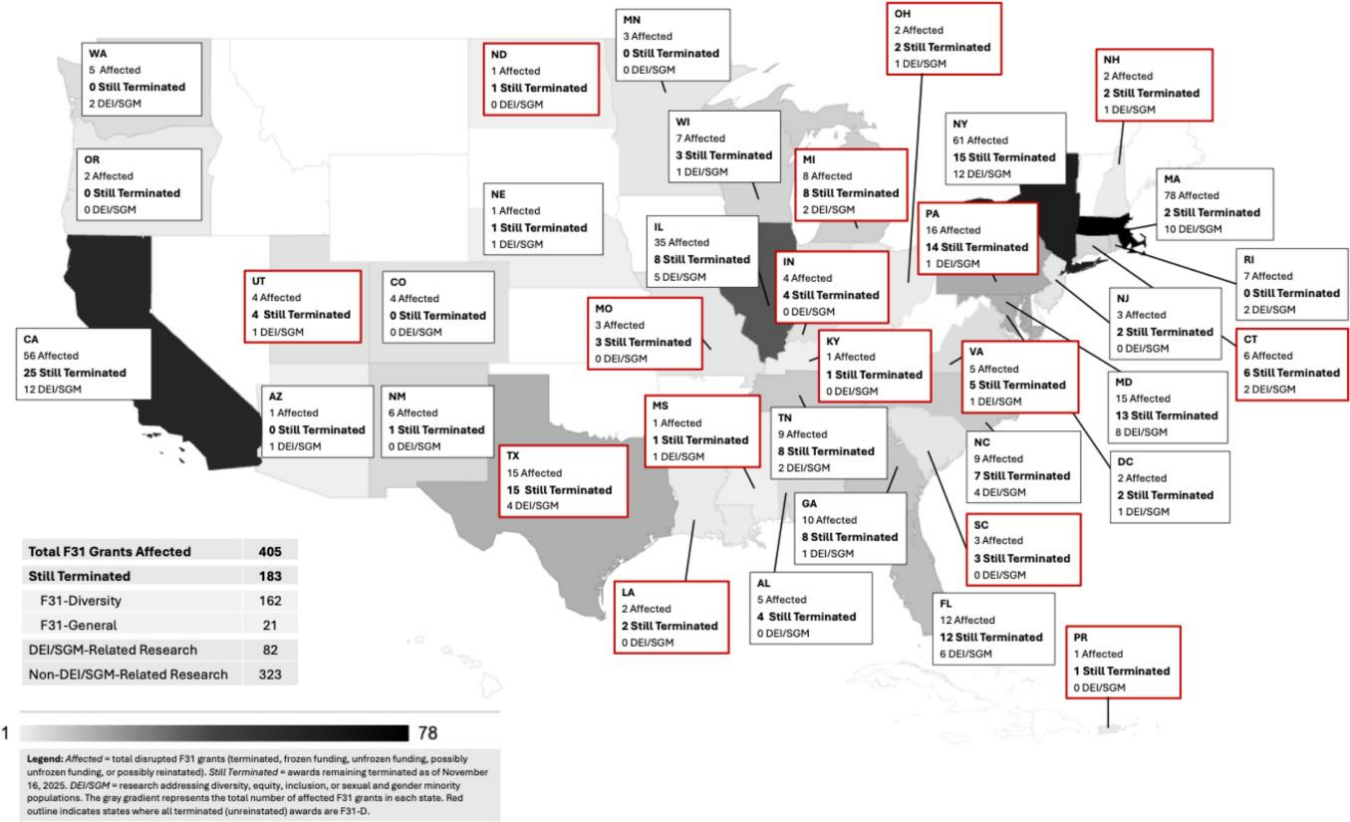
Termination Status of disrupted NIH F31 grants and DEI/SGM-related research by state.

Using the keyword list defined by *Grant Witness* (Table S1), we also found that 191 (47%) F31 grants contained race or DEI-related flagged words, 67 (17%) gender/LGBTQ + words, and 29 (7%) HIV-related words. Our manual abstract review indicated that most terminated projects were not focused on DEI or SGM populations (*n* = 323) and instead examined broader areas related to obesity, Alzheimer's disease, or immune function. Fewer terminated projects (*n* = 82) explicitly focused on DEI- and SGM-related topics, including disparities in HIV prevention, substance use, and access to behavioral health services among women living with HIV, Black and Latino SGM populations, and immigrant communities. To visualize these patterns, we mapped the counts of affected grants by state and overlaid topic-area categories (ie, DEI/SGM related) (Figure 1). In states with only 1 or 2 terminated grants, such as Mississippi, New Hampshire, and Ohio, all affected grants were held by underrepresented scholars, resulting in the loss of trainees conducting both DEI/SGM-focused and non-DEI research. Although most terminated projects were in non-DEI/SGM-related areas (areas one might expect to be prioritized for reinstatement), reinstatements remained limited across states, indicating that terminations persisted across both F31-G and F31-D mechanisms regardless of research topic.

## Discussion

Our analysis highlights that the 2025 F31 terminations were widespread and unevenly concentrated among diversity-promoting fellowships, with considerable geographic clustering in the South and Midwest, and limited reinstatements even for non-DEI/SGM research. Despite partial reversals, many predoctoral trainees remained terminated or frozen, reflecting ongoing disruptions and uncertainty across both F31 general and diversity mechanisms and research areas. Consistent with recent work showing disproportionate impacts on underrepresented minority scholars,^28^ our findings reveal that these disruptions extend to predoctoral trainees.

Emerging literature documents the short-term impacts and potential long-term implications of the recent 2025 abrupt funding suspensions on the predoctoral training pipeline.^29^ For example, in a report from University of California, Los Angeles, predoctoral students identified consequences along the F31 training pathway from pre-submission (eg, confusion about which F31 mechanism remains viable), submission (eg, concerns about whether the application will be reviewed, withdrawn, or rejected), grant termination (eg, distress about funding continuity, dissertation completion, timely graduation), and grant reinstatement (eg, uncertainty about release of funds).^30^ Court filings similarly documented that sudden grant terminations placed students “who often live paycheck to paycheck”^31^ (p. App.225) at immediate risk of losing housing and income, jeopardizing their ability to meet basic needs. Even researchers whose grants were restored following recent court rulings describe ongoing confusion and delays concerning the release of their funds,^32^ which indicates that even when funding decisions are reversed, challenges remain to ensure that reinstatement orders are fully implemented.^32^ Longer-term, there are likely to be^30,31,33,34^ generational impacts on the local and national public health research workforce, infrastructure, and innovation due to the discontinuation of training grant supports like the F31. Such disruptions may damage the pipeline of future researchers whose options for training are dwindling. Reduced access to training opportunities may diminish competitive leverage for subsequent funding and career advancement, leading some to leave academia altogether.^30,34^

These findings suggest a need for policies that better protect predoctoral trainees. Informed by emerging literature,^35^ we propose the following recommendations. First, the NIH should establish legal safeguards (eg, mid-year termination bans and emergency trainee support funds during grant disruptions to support trainees as they transition out of lost funding) for the existing general F31 mechanism and T32-training programs, which could shield students from political shifts and provide legal recourse for obtaining the remaining funds they were promised when funding is pulled overnight.^33,36^ Second, academic institutions could establish bridge funding mechanisms^37^ and develop cross-institutional alliances where schools share resources (and available mentors with funding) to insulate students from funding cuts. Third, states, especially those that lost significant research capacity from grant terminations, could develop state-level public-private partnerships modeled after the Georgia Research Alliance^38^ and expand these frameworks to strengthen local research infrastructure and support early-career researchers during periods of funding disruption. Fourth, university departments with doctoral programs could provide students with guidance and financial support to access litigation,^21^ appeals processes, and legal information networks to facilitate their participation in these types of proceedings. Fifth, students should be mentored in diversifying funding streams;^39^ although foundation and philanthropic funding are already highly competitive, institutions could provide training on how to write successful grant applications and encourage students to apply for local and national scholarships that engage in partnerships with local communities, relationships that are more important now than ever. Lastly, students should be provided a platform, as was similarly done by the American Journal of Public Health,^40^ to provide testimony and consult with policymakers on how policies shape their education, so that the public can fully understand their impact.

## Conclusion

This brief report includes an analysis of publicly available U.S. NIH-F31 terminations to illustrate how these disruptions affect trainees and proposes policy safeguards. Our analysis of *Grant Witness* data demonstrated that F31 terminations were widespread and unevenly distributed across states and mechanisms, with F31-Diversity trainees experiencing higher proportional rates of termination and fewer reinstatements. As the funding landscape continues to evolve (and even as some funding decisions are reversed), systematic monitoring and evaluation of grant disruptions are essential to assess the downstream effects of grant terminations on predoctoral research trajectories and scientific priorities.

## Supplementary Material

qxag065_Supplementary_Data
