# Supplementary material for: Patterns and implications of 2025 NIH-F31 grant terminations for the predoctoral training pipeline
Source: Health Aff Sch. 2026 Mar 18;4(3):qxag065. doi: 10.1093/haschl/qxag065 (PMC13032874; doi:10.1093/haschl/qxag065)
Supplement: qxag065_Supplementary_Data [file qxag065_supplementary_data.zip › Supplementary Table 1.docx]

**Supplementary Table 1.** Keywords Used to Classify F31 Grant Abstracts as DEI/SGM and non-DEI/SGM-Related

| **Category** | **Keywords** |
| --- | --- |
| DEI/SGM***** | anti-racism, barrier, barriers, bias, culturally appropriate, discrimination, discriminatory, disparity, diverse backgrounds, diverse communities, diverse community, diverse group, diversify, diversifying, diversity, enhance the diversity, enhancing diversity, equitable, equity, ethnicity, excluded, hate speech, health disparity, health equity, immigrants, implicit bias, implicit biases, inclusion, inclusive, inclusiveness, inclusivity, increase diversity, increase the diversity, inequalities, inequality, inequitable, iniquities, inequity, injustice, intersectional, intersectionality, marginalize, marginalized, minorities, minority, multicultural, oppression, political, prejudice, privilege, promote diversity, promoting diversity, race, and ethnicity, racial, racial diversity, racial identity, racial inequality, racial justice, racially, racism, segregation, social justice, stereotype, stereotypes, systemic, underrepresentation, underrepresented, underserved, female, females, gender, identity, genders, LGBT, LGBTQ, non-binary, nonbinary, pregnant people, pregnant person, pregnant persons, sex, women, women and underrepresented, affirming care, assigned at birth, assigned female at birth, assigned male at birth, gender diversity, gender identity, gender affirming care, transgender |
| Not specifically DEI/SGM****** | bioinformatic, biomechanic, cellular, chromatin, computational, DNA, epigenetics, genetics, genomics, immunity, laboratory, mechanistic, metabolic, metabolism, microbiome, microbiology, mitochondria, molecular, neurogenetic, physiology, protein, proteomics, RNA, sequencing, signaling, stem cell, transcriptional, transcriptomics, vaccine development |

*****Keywords were derived from *GrantWitness*.

******Terms reflected basic, laboratory-based, or mechanistic scientific research without explicit reference to health disparities or underserved populations in the abstract.
